# Analysis of Spatial Point Patterns in Nuclear Biology

**DOI:** 10.1371/journal.pone.0036841

**Published:** 2012-05-16

**Authors:** David J. Weston, Niall M. Adams, Richard A. Russell, David A. Stephens, Paul S. Freemont

**Affiliations:** 1 Department of Mathematics, Imperial College London, London, United Kingdom; 2 Heilbronn Institute for Mathematical Research, University of Bristol, Bristol, United Kingdom; 3 NIHR Biomedical Research Centre for Ophthalmology, Moorfields Eye Hospital NHS, Foundation Trust and UCL Institute of Ophthalmology, London, United Kingdom; 4 Department of Mathematics and Statistics, McGill University, Montreal, Canada; 5 Division of Molecular Biosciences, Imperial College London, London, United Kingdom; Institut de Génétique et Développement de Rennes, France

## Abstract

There is considerable interest in cell biology in determining whether, and to what extent, the spatial arrangement of nuclear objects affects nuclear function. A common approach to address this issue involves analyzing a collection of images produced using some form of fluorescence microscopy. We assume that these images have been successfully pre-processed and a spatial point pattern representation of the objects of interest within the nuclear boundary is available. Typically in these scenarios, the number of objects per nucleus is low, which has consequences on the ability of standard analysis procedures to demonstrate the existence of spatial preference in the pattern. There are broadly two common approaches to look for structure in these spatial point patterns. First a spatial point pattern for each image is analyzed individually, or second a simple normalization is performed and the patterns are aggregated. In this paper we demonstrate using synthetic spatial point patterns drawn from predefined point processes how difficult it is to distinguish a pattern from complete spatial randomness using these techniques and hence how easy it is to miss interesting spatial preferences in the arrangement of nuclear objects. The impact of this problem is also illustrated on data related to the configuration of PML nuclear bodies in mammalian fibroblast cells.

## Introduction

**Figure 1:**
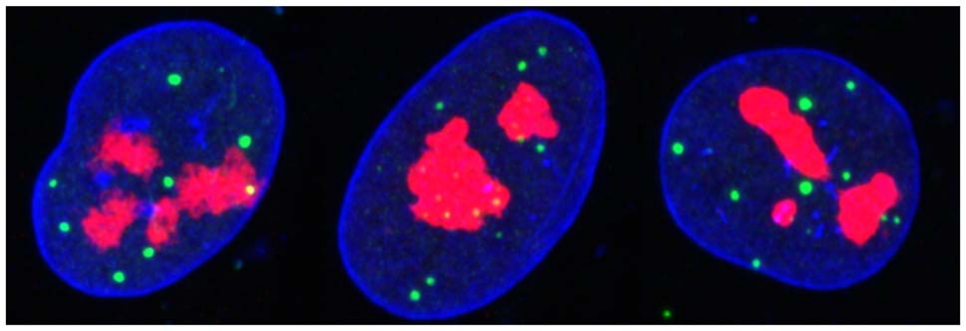
2D projections of MRC5 nuclei imaged using CSLM. Objects of interest have been stained using immunofluorescence. The PML NBs are stained green, the red staining are nucleoli and DNA on the boundary of the nucleus are stained blue.

The eukaryotic cell nucleus is a membrane-bound organelle that performs vital functions in regulating, translating and replicating the cell’s genome. The nucleus contains distinct structures comprising assemblies of macromolecular complexes referred to as nuclear compartments [1]. Examples of such compartments include splicing speckles, chromosome territories, nucleoli and PML nuclear bodies [2]. Currently the most popular approach to acquiring images of the internal structure of the nucleus is to use confocal laser scanning microscopy (CSLM) [3], see Materials and Methods for more detail. Examples of CSLM acquired images are shown in Figure 1 which displays a number of imaged cell nuclei in 2D projection.

There is considerable interest in determining to what degree the spatial organization of these compartments affects the function of the nucleus, [4], [5] and consequently such behavior as cell division and cell growth. A typical approach for exploring the spatial preference of compartments involves an exploratory spatial hypothesis test to determine if the observed point pattern is consistent with the simplest spatial model: complete spatial randomness (CSR), that is, a homogeneous spatial Poisson process. Biologically, this is not an interesting model – corresponding to a compartment that has no spatial preference and no self-interactions. While this is biologically implausible, it is a good starting point for formal analysis, as will be discussed later. The concern of this paper is to demonstrate that standard data analysis approaches for exploring spatial preference, particularly for compartments which manifest as few objects per nucleus, are prone to overlook interesting preferences.

To demonstrate this, we have generated synthetic nuclear images, exploring the diversity of nuclear shape and using specific spatial processes. We have investigated the statistical power of a number of hypothesis tests procedures against specific alternatives to CSR. This synthetic data is used to demonstrate that looking at replicate nuclear images one at a time can lead to equivocal results. We also show that simple procedures for combining information across images and constructing a test statistic are potentially unreliable. Our results add a cautionary note to the routine application of quantitative spatial analysis methods, which have the potential to miss interesting spatial preferences and relationships in CSLM images.

### Problems Associated with Spatial Point Pattern Analysis

In this section, we provide an overview of quantitative reasoning about nuclear architecture and the difficulties that can arise. This paper is primarily concerned with the inadequacy of simple procedures to reveal complicated spatial preferences in replicate nuclear compartment point patterns. Thus it is assumed that the data acquisition and pre-processing steps mentioned above have been successfully applied, yielding a collection of processed images. In this case, we presume the processing has provided 2 things: First, a representation of the shape of the nucleus boundary; second, the 3D spatial coordinates (the *point pattern*) of the target compartment. Note that the compartments themselves also have extent meaning that they will be represented by more than one pixel location within the image stack. We are concerned with compartments that can reasonably be represented as a spatial point pattern, which means that, for example, the centroids of the compartment of interest yield the point locations.

Given such processed data, the main problem is making inferential statements given a collection of point patterns derived from replicate nuclei images, with the objective of identifying interesting and potentially informative spatial arrangements. It is common in the literature [6] to perform one or more hypothesis tests under the null hypothesis of CSR. CSR corresponds to a spatial Poisson process [4], the simplest and most trivial case in which the points are placed independently, and exhibit the same average number of points in any fixed sized region within the nucleus. Rejecting the hypothesis of CSR is evidence that the generating process is more complicated and hence potentially interesting. Diggle argues that rejecting the hypothesis of CSR should be used as a trigger for more formal analysis [4]. It is however frequent in the nuclear biology literature to stop at this point, simply denoting these non-CSR patterns as “non-random” 7,8. This is an unfortunate misnomer, incorrectly suggesting that the process is non-stochastic, when the evidence only supports that it is a non-CSR stochastic process.

A common approach is to analyze the spatial point pattern derived from each nucleus in isolation from one other, [9]. This will typically result in equivocal conclusions, with a certain proportion of nuclei whose spatial arrangements successfully reject the null. It is difficult to interpret this result in order to make a quantitative statement concerning whether a non-CSR spatial arrangement exists within this collection of nuclei.

An alternative approach is to artificially “inflate” the effective number of points in the pattern. One way to achieve this is by combining statistics gathered from the replicate data prior to performing the hypothesis test. There can however be considerable variation between nuclei sizes and shapes and the number of compartments of interest contained within them. To make the statistics extracted from multiple nuclei commensurate some appropriate transformation is required. In [10] statistics based on distances are used, for each nucleus the distances are normalized using the largest inter-point distance within the nucleus. Later, it is shown that such a simple normalization does not properly respect the heterogeneity of nuclear shape. In [11], data are transformed to the same coordinate system, using a manually located landmark and manually assessed orientation.

Some choice of alternatives to the null hypothesis of CSR includes processes for which the point locations are closer together than expected. Simple patterns of clustering that are biologically interesting include points being preferentially located near the nuclear boundary, points clustering near the center and points clustering around the poles of the nucleus. An alternative to clustering are patterns where the points are further away from each other than expected under CSR. Finally, spatial relationships between different compartment types, for example a preference for one compartment type to be close to another, is another potentially interesting spatial arrangement.

For compartments that manifest as small number of objects per cell the statistical power of such tests against these specific alternatives is likely to be low. Indeed this is explicitly illustrated in our results section.

### Characterizing Nuclear Compartment Locations

In the previous section generic difficulties related to point pattern analysis to nuclear architecture were described. This section considers some specific statistical procedures. Our objective is not to thoroughly review all approaches but to describe popular modern procedures.

Since the configuration of compartments in any individual nucleus is different, there is a need to appeal to quantitative procedures to make formal statements about the character of the configuration. As noted above, there is a preference in the literature to refer to a pattern as random, or non-random. While this is an imprecise characterization, such a determination requires a statistical test procedure. For compartments that can reasonably be treated as a point pattern, a realization of a stochastic point process [4], there are a number of possible approaches. By far the most popular approach deployed in the literature uses exploratory hypothesis testing, based on distance relationships observed among the points [6]. The tests proceed under the null hypothesis of CSR. The simplest tests are based on so-called point pattern *summaries*, a single number representing the inter-point distances. More refined approaches use so-called point pattern *characteristics*, functions of inter-point distances. The empty space function, or F-function, 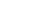, is based purely on distances between points and consequently is invariant to rotation and translation – implicitly assuming that the generating process is stationary and isotropic. In our case, this function is defined over a region R that contains the point pattern in question. 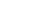 is a cumulative distribution function and is equal to the proportion of the region R that lies within a distance 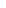 of any point in the point pattern. There are a number of alternative distance based functions, see for example [12]. The F-function appears to have better discriminatory power for spatial organization [13]. For simplicity of exposition, the F-function is the preferred choice in this paper.

### Strategies for Combining Replicates

Reporting the results of individual tests, such as those described above, for replicate image data is likely to lead to equivocal results, particularly for compartments that have a low expected number of occurrences. Some nuclei will reject the null hypothesis while others will fail to so do. This raises the question of how to reason about the *population* of nucleus images given such results. One approach is to treat the replicate images as a population, and aggregate information across different cells. An example of aggregating imaging information across cells is described in [6]. Since the F-function is not scale invariant, a simple approach to normalize the F-function is to divide inter-point distances by the maximum length obtainable within the cell. The domain for each cumulative F-function curve will thus be from zero to one inclusive. The population F-function curve is then produced by averaging the normalized F-functions from each nucleus at each distance *r*.

One recent approach that can extend the analysis to the population level is described in [13], where a quantity denoted the *spatial distribution index* (SDI) is defined. For a population of point pattern realizations drawn from a CSR process, the corresponding SDI values have the property that they are distributed uniformly over 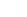. The full details for calculating SDI can be found in the Materials and Methods section.

There are two approaches to performing a Monte Carlo significance test based on summary functions such as F(*r*), these are often denoted *envelope* and *Goodness-of-fit* [14]. For our experiments we examine both approaches, the F-function Test (both individual and aggregate-distance) are examples of the former and SDI is an example of the latter. Briefly, for Goodness-of-fit tests, a single number is used to characterize the discrepancy from a reference summary function whereas for envelope based tests discrepancies across a range of values of *r* are examined. Envelope based tests are considered more problematic due to difficulties in determining the significance level [14].

**Figure 2:**
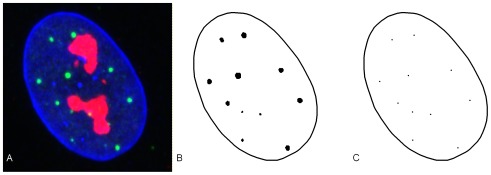
2-D Illustration of the extraction of PML locations from image data. A) Original raw image B) The boundary of the nucleus envelope and the PML bodies are segmented from an image. C) Each PML body is replaced by its center of gravity.

**Figure 3:**
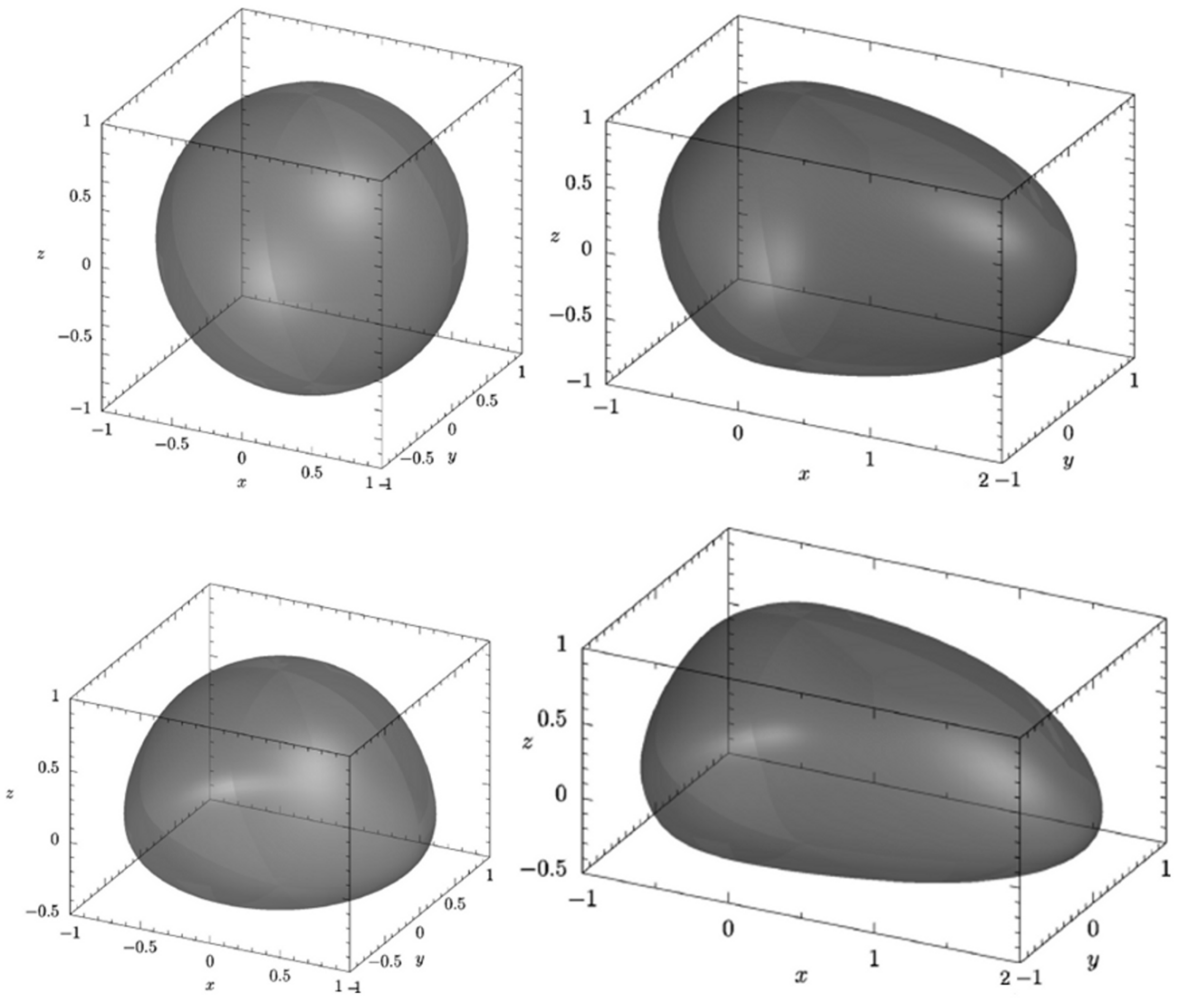
Cell-like synthetic boundaries. The top row are a sphere and a more ellipsoidal shape, on the bottom row are the same shapes but with their underside flattened. Deformed instances from each of these 4 shape classes are used in the analysis of synthetic data. This collection is referred to as the thick set. A second collection denoted the thin set differ by having a one fifth the height (z –axis).

### PML Compartments

To illustrate the character of the problem outlined above, we will use as an example the spatial analysis of the Promyelocytic Leukemia nuclear body (PML NBs) compartment [9], [15], [16]. PML NBs have been linked to a number of roles including response to DNA damage and apoptosis, for a review see [17]. There have been several studies that suggest associations with other compartments [9]. Such associations are used to imply functional relationships and involvement in nuclear pathways. However, the diverse roles of PML bodies are still poorly understood [18].

In mammalian cells, the PML compartment manifests as a number of small (typically less than 1 micron) nuclear bodies (NB). These are PML protein aggregates containing a number of PML isoforms and a collection of other resident proteins including SUMO, sp100 and Daxx, [9]. Figure 1 displays the 2D projection of three mammalian fibroblast cells, immunoflourescently labeled, with PML bodies in the green channel. The simplest question one may ask in relation to the spatial positioning of the PML NBs in these nuclei is determining whether they are consistent with a CSR process, i.e. whether the PML NBs placed independently and such that the expected number of bodies is the same everywhere. As an aside, it is worth noting a general point regarding the simple inspection, or quantitative analysis, of 2D projection data. Such inspection ignores the true 3D structure and has the potential to suggest relationships that are merely artifacts of projection. However the thinness of cell nuclei from many cell lines mitigates this issue.

**Figure 4:**
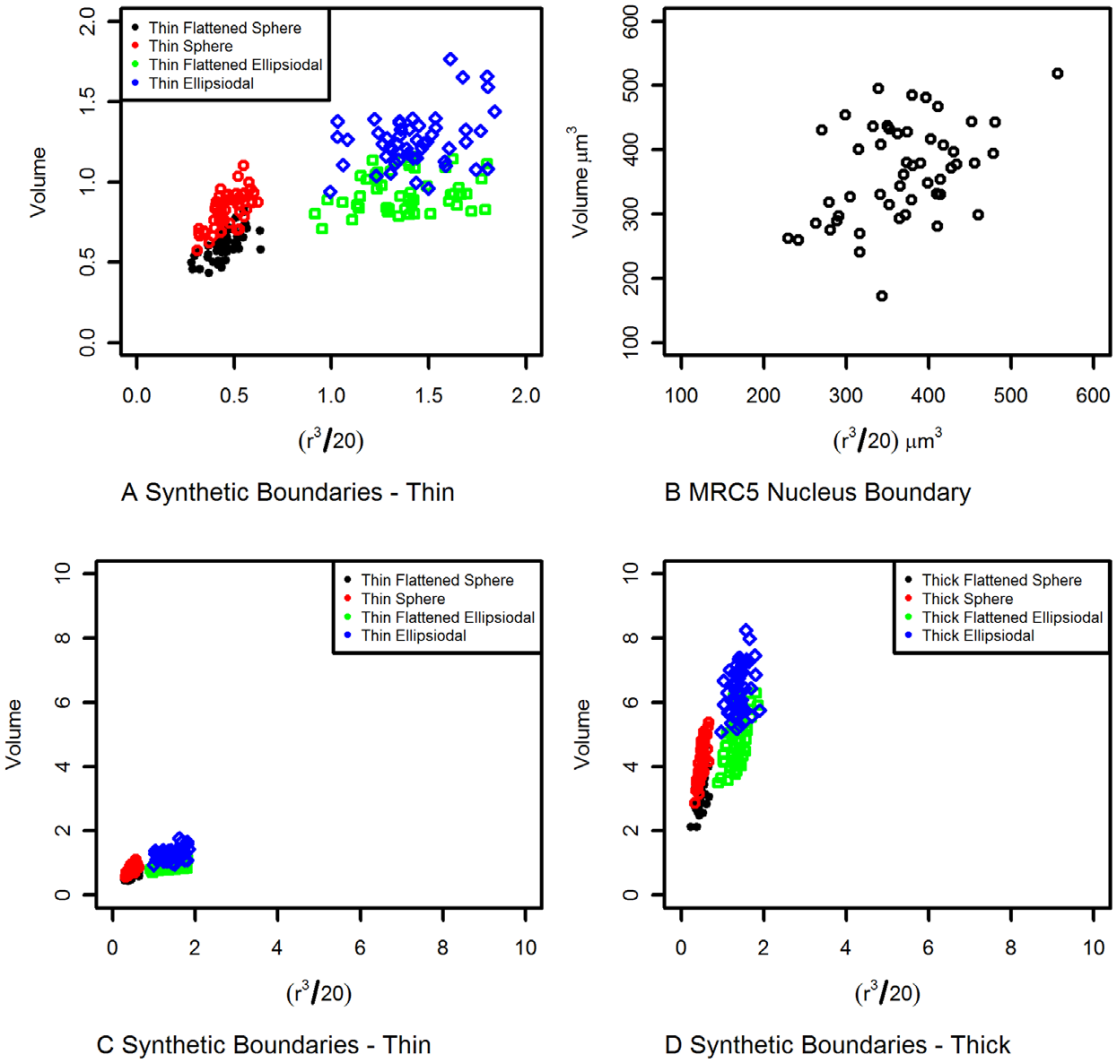
Demonstrating Boundary Shape Heterogeneity. The figures show how the cube of the maximum inter-point distance within a boundary varies with the volume enclosed by the boundary. If all the boundaries had the same shape then all the points on each graph should fall on a straight line. A) shows 50 instances from each of the 4 thin synthetic boundary shapes. It demonstrates that the scatter within each shape class is much smaller than between shape classes. B) Nucleus envelopes from MRC5 dataset demonstrating scatter. C) shows the same data as A) but on a different scale, so that it can be compared with D), where D) shows 50 instances from each of the 4 thick synthetic boundary shapes shown in Figure 3.

**Figure 5:**
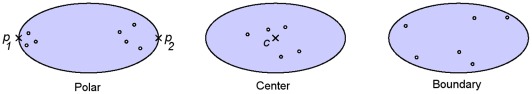
2-D Illustrations of the alternative spatial point processes used in the construction of the synthetic data. Each of the three alternatives has a clear spatial preference. The challenge for spatial analysis is to identify such preference when the average number of points is low and potentially difficult to distinguish from CSR.

**Figure 6:**
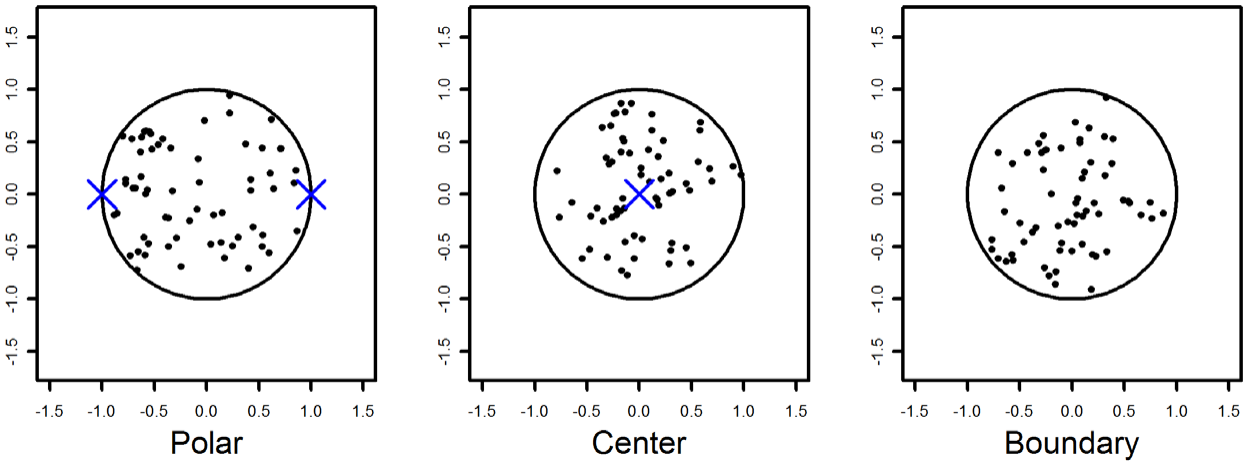
2-D Projections of instances for each of the alternative spatial point processes used in the construction of the synthetic data. The ordering of these patterns and the crosses (highlighted in blue) correspond to those in Figure 5. These patterns each contain 64 points and are far more difficult to identify through visual inspection than the schematic illustrations shown in Figure 5.

## Results

### Dataset Description

The following experiments are designed to highlight difficulties in applying spatial point pattern analysis tools to determine whether there is interesting spatial preference, especially in the case of patterns generated by compartments that manifest few objects per nucleus. Both a real and a synthetic dataset are examined. The real data, described below, is an example of a point pattern consisting of a small number of objects, namely PML NBs, described above. Whether the objects follow CSR and if not what alternative spatial point process adequately describes the spatial arrangement is still a subject of active research. The motivation for using synthetic image data is to have access to spatial point patterns that follow known point processes. This ground truth is used to measure the performance of the analysis methods described above. The following section describes the data in detail, where we also include an analysis of the extent of heterogeneity in the nuclear boundaries. This is especially pertinent when an aggregation method is used. For the synthetic data, spatial point patterns are generated using specific alternatives to CSR.

**Figure 7:**
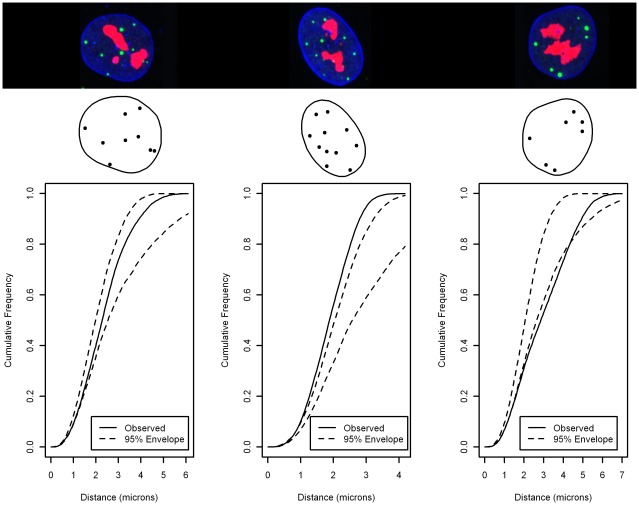
Three MRC5 nuclei with PML locations and their corresponding F-function Test. The images are a 2-D projection but the analysis is performed in 3-D. If the observed F-function (the continuous curve) is wholly contained within the CSR envelope (the dotted curves) then the pattern is consistent with CSR (the null hypothesis). The middle nucleus rejects the null and is demonstrating PML locations that are further apart than would be expected by CSR. The right-most nucleus also rejects the null and is demonstrating locations that are more tightly clustered than would be expected by CSR.

#### MRC5 data

The real data consists of 50 images of nuclei from MRC5 cells extracted from an asynchronous cell culture, examples are shown in Figure 2. The data is processed to extract the locations of nucleus boundary and PML bodies, using the methods in [2]. For a specific nucleus, the left image in Figure 2 shows the extracted boundary and the PML compartments viewed projected onto the xy-plane. The PML compartments have an extent, so to reduce the PML compartments down to a point pattern; we replace the extracted PML compartments with their respective centers, shown in the right-hand side image in Figure 2.

The number of PML compartments extracted from each image varied from 5 to 19. In total, there are 482 NBs in the collection of 50 images.

**Figure 8:**
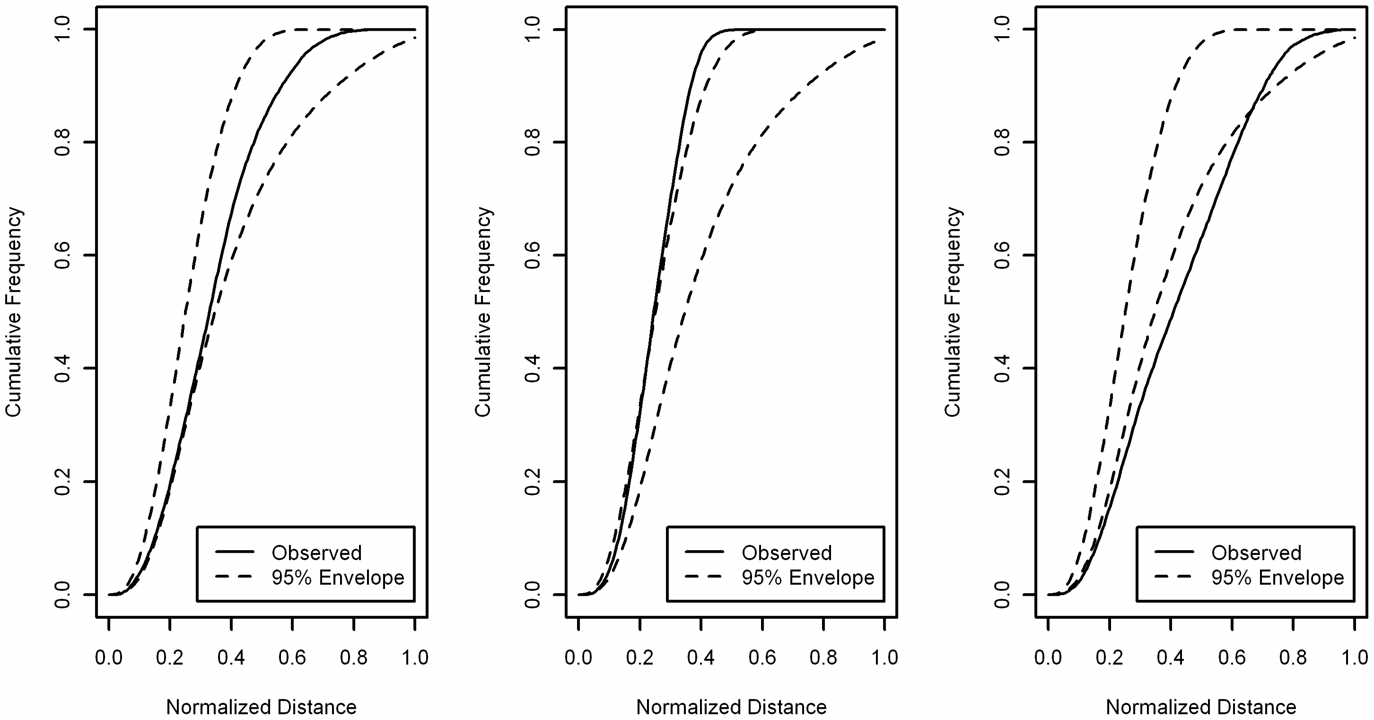
Modified *Individual* F-function Test using Aggregated null envelope for 3 MRC5. The distance standardized F-functions of the PML NB locations for the nuclei shown in Figure 1. Although for these particular cells we make the same decision as Figure 7, the result over the dataset is different; fewer nuclei fail to reject the null. This is likely due to the shape heterogeneity of the nucleus envelope, evidence for which is provided by the investigation of synthetic data, see Figure 11.

#### Construction of synthetic data

We propose to investigate point patterns contained within three dimensional boundaries that have a broadly cell-like shape. We note that the boundary of the nuclei shown in Figure 1 exhibit considerable variability. Our intention is not to make plausible synthetic boundaries for the nucleus rather we wish to show how large differences in boundary shape can affect the outcome of any analysis when simple aggregation procedures are used. More sophisticated models for generating boundaries can be found in [19], [20]. The boundaries used are based on combining four ellipsoids in a piecewise fashion (see Materials and Methods).

**Figure 9:**
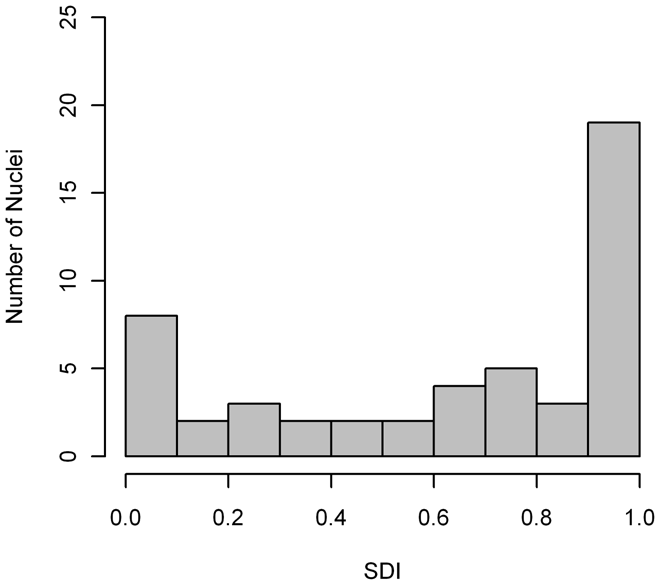
Histogram of SDI using F-function for MRC5 PML NB locations. For a CSR process we would expect the histogram to be approximately uniform, however we see concentrations in both the low and high values of SDI indictaing that there is a spatial preference for PML NBs.

Two collections of shapes are investigated. The first collection is shown in Figure 3, where the top row are a sphere and a more ellipsoidal shape, on the bottom row are the same shapes but with their underside flattened. Fifty instances of each of these shapes are generated by perturbing the parameters specifying each shape, the details of the scale of the perturbation can be found in Materials and Methods. The second collection differs only in their height. They are thinner by a factor of 5 and have a commensurate height to maximum length exhibited in the cell nuclei of the MRC5 dataset. We refer to these datasets as the *thick* and *thin* datasets respectively.

#### Analysis of object boundary shapes

Aggregating over replicate nuclei would be considerably simplified if they had the same shape. However, it is typically the case that the nucleus exhibits large variation in the shape of the boundary. Figure 4(A,C-D) shows how the volume contained within the boundary of each instance from the synthetic data varies with the cube of the maximum inter-point distance within each instance. If the boundaries were simply scaled versions of each other then the points on the graph would follow a line. As expected, there is substantial scatter between instances of the four shape types. Figure 4(A-B) shows the plot for the thin dataset and the corresponding plot for the real data. The real data shows substantial scatter, suggesting the boundaries also have substantial differences in shape. This is likely to cause problems for any method that simply standardizes according to the size of boundary in order to aggregate results. Of course different cell lines will exhibit different degrees of boundary shape heterogeneity and consequently the power of the tests based on aggregation will differ.

**Figure 10:**
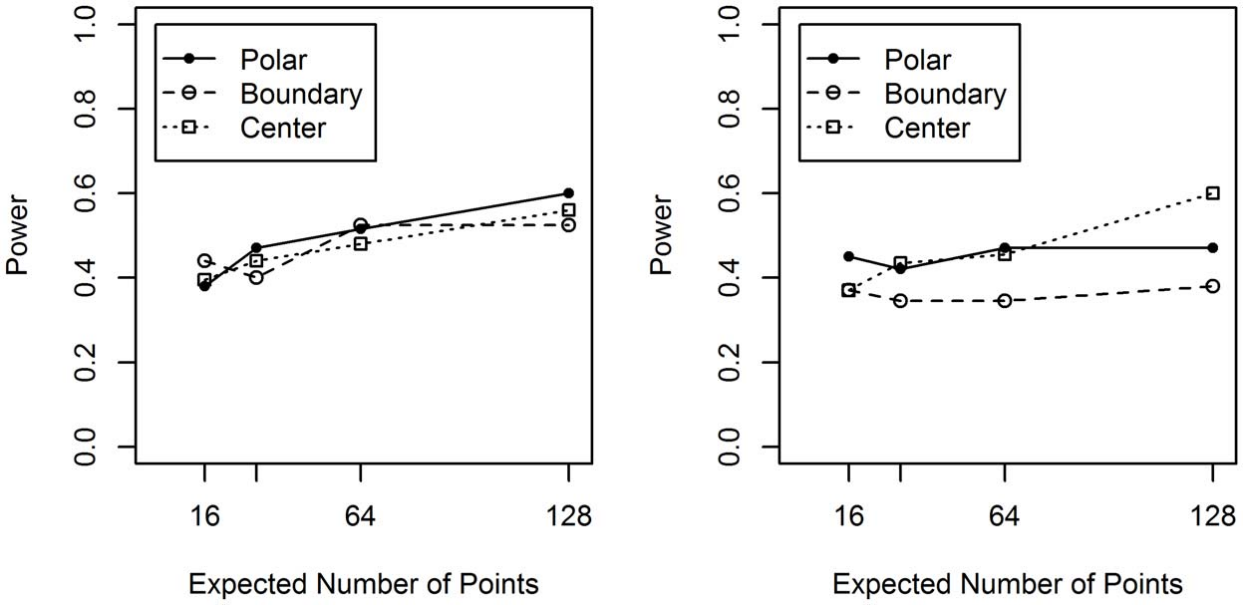
*Individual* F-function Test for synthetic datasets. The leftmost figure corresponds to the thick dataset and the rightmost the thin dataset. For each of 200 synthetic nuclei, in each dataset, an F-function Test is performed, where the null hypothesis is the spatial point pattern has been drawn from a CSR process. The spatial point patterns generated for these all the synthetic instances are from alternatives to CSR, hence the power of the test is equal to the proportion of instances that reject the null hypothesis.

**Figure 11:**
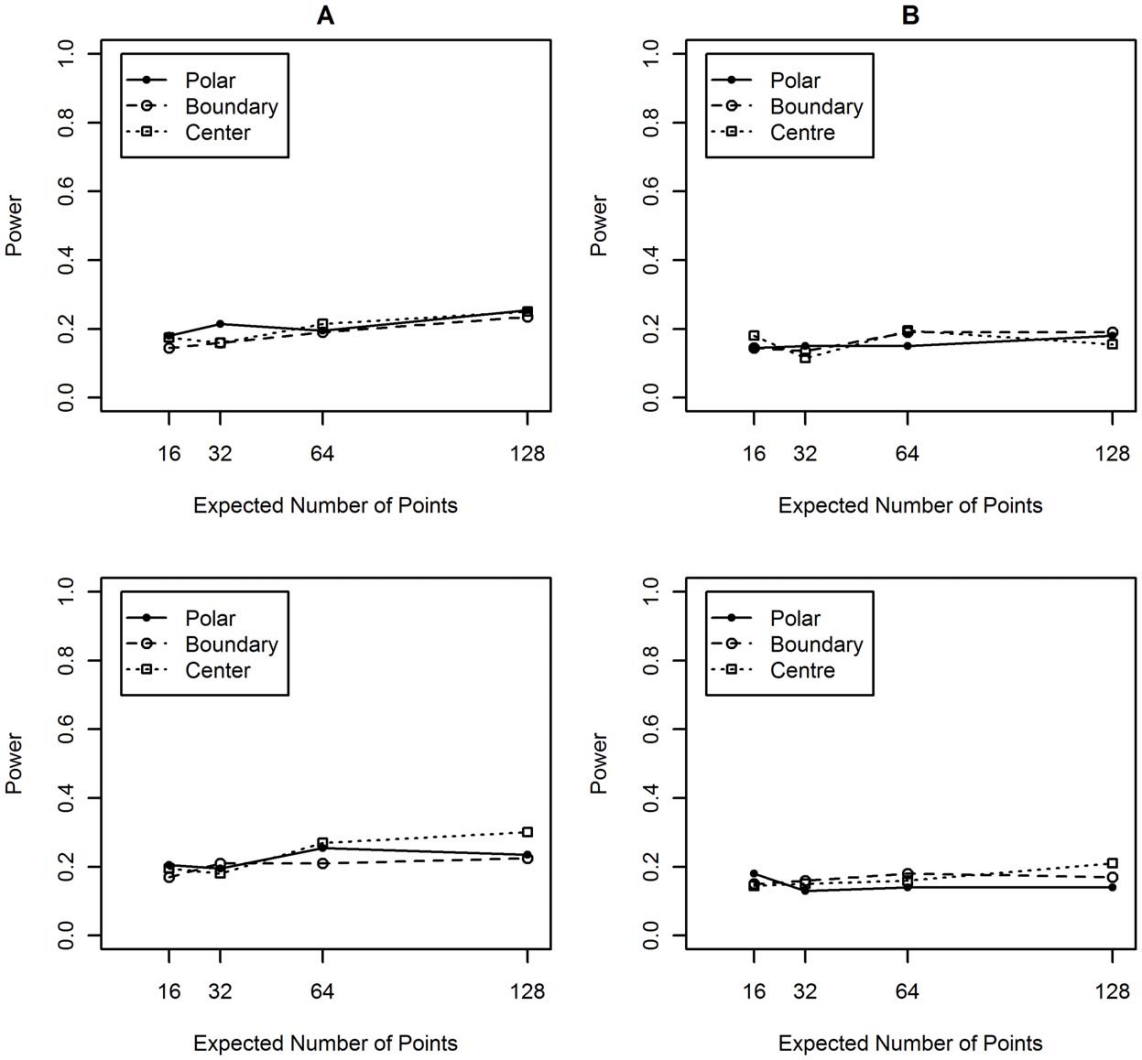
*Modified Distance* F-function Test. Performing an F-function Test over 200 synthetic nuclei instances similar to that shown in Figure 10. The top row are results for the thin shape collection and the bottom row are the results for the thick shape collection. In the first column the null distribution is aggregated over: A) instances from the same shape class and B) aggregating over all instances. Aggregation over all shape classes (B) clearly reduces the power of the test. Even aggregating within a shape class (A) decreases performance relative to individual analysis.

#### Alternatives to complete spatial randomness

Inside each instance of a cell shape, we generate points following spatial point processes other than CSR, as follows. Three alternative compartment spatial preference models are investigated. These are denoted *Polar*, *Center* and *Boundary* and describe spatial point processes that produce patterns that locate themselves preferentially near the poles of the nucleus, the center of the nucleus and its boundary, respectively. Details of the construction of these processes are described in the Material and Methods. Figure 5 shows an illustration of each of these processes. For clarity these examples are displayed with a two-dimensional boundary, however the experiments are all performed using three-dimensional shapes. This choice of alternatives seems natural for the types of configuration one might expect in a biological system, [21], [22], [23]. Note that, while the real data example is concerned with PML NBs, this simulation will be relevant to any compartment that manifests as few objects per nucleus. Actual generated examples of these three processes projected in 2D are shown in Figure 6. Note that, first it is difficult to distinguish these processes visually, and second the patterns of concern typically exhibit fewer objects, making the problem more difficult. A further complication that Figure 6 ignores, central to this paper, is the impact of cell shape variability.

**Table 1:** Two-sided K-S test statistics for SDI using the F-function.

Thin						
	Polar		Boundary		Center	
Expected number of points	D	p-value	D	p-value	D	p-value
16	0.097	0.046	0.088	0.090	0.163	4.85E-05
32	0.228	1.86E-09	0.063	0.405	0.178	6.27E-06
64	0.206	8.49E-08	0.059	0.490	0.261	2.93E-12
128	0.314	<2.2E-16	0.066	0.348	0.399	<2.2E-16
**Thick**						
	**Polar**		**Boundary**		**Center**	
**Expected number of points**	**D**	**p-value**	**D**	**p-value**	**D**	**p-value**
16	0.157	1.05E-04	0.134	0.002	0.116	0.009194
32	0.281	3.84E-14	0.066	0.348	0.156	0.000118
64	0.277	9.37E-14	0.076	0.198	0.218	1.11E-08
128	0.390	<2.2E-16	0.129	0.003	0.293	2.44E-15

For spatial point patterns generated by each type of alternative to CSR deployed, and for increasing average number of points, the summary statistic from the SDI, D, and its corresponding p-value are shown. Total number of synthetic nuclei is 200.

**Figure 12:**
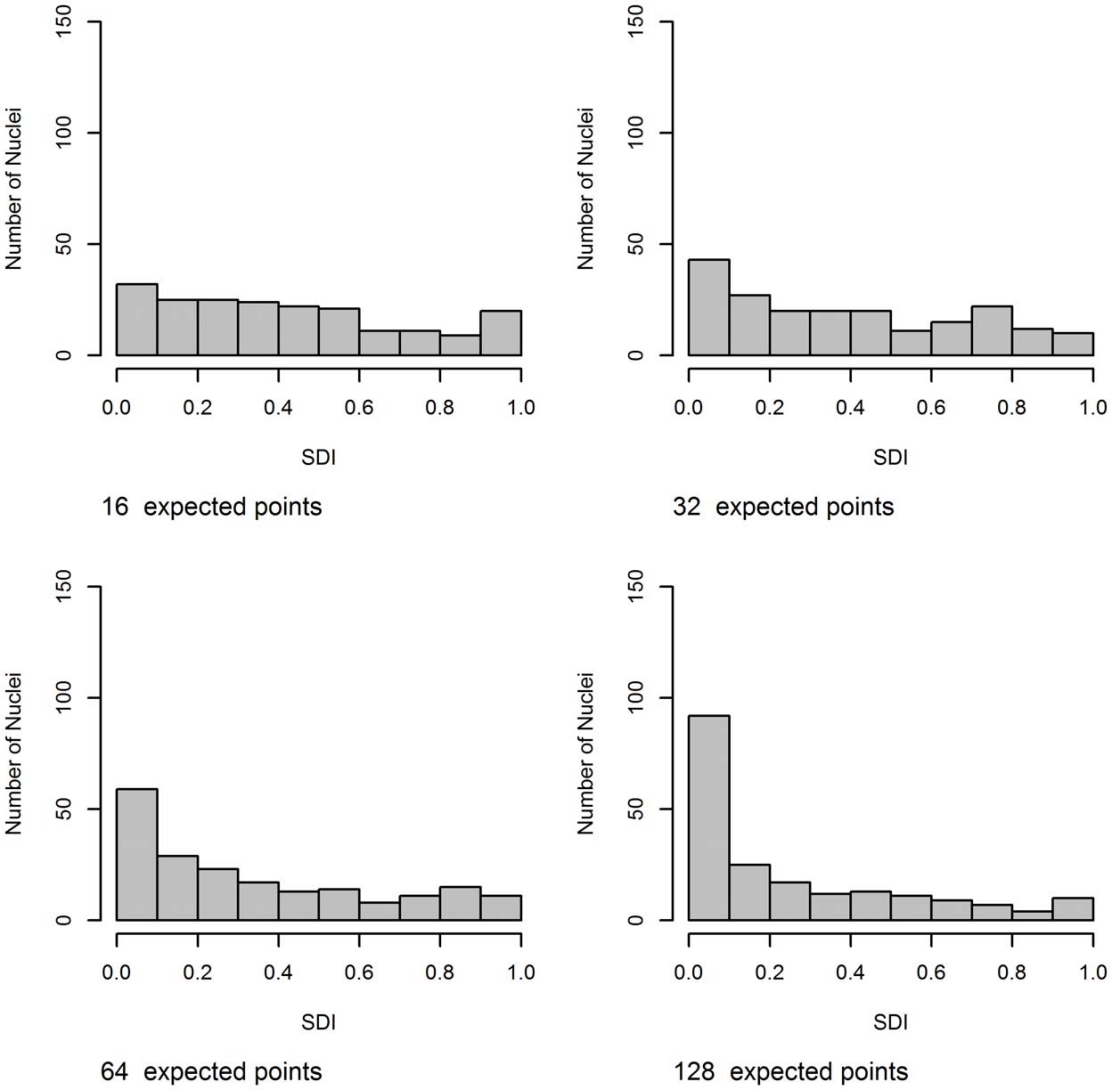
Histograms of SDI using F-function for the center alternative for the thin dataset with increasing number of expected points. The expected number of points increases top left to bottom right. For a CSR process the histogram should be uniform. For 16 expected points it is difficult to distinguish the histogram from uniformity. However, as the average number of points increases we see an increase in the non-uniformity of the histogram. In particular we see a concentration for low SDI values (which is what would be expected for points that are clustered).

**Figure 13:**
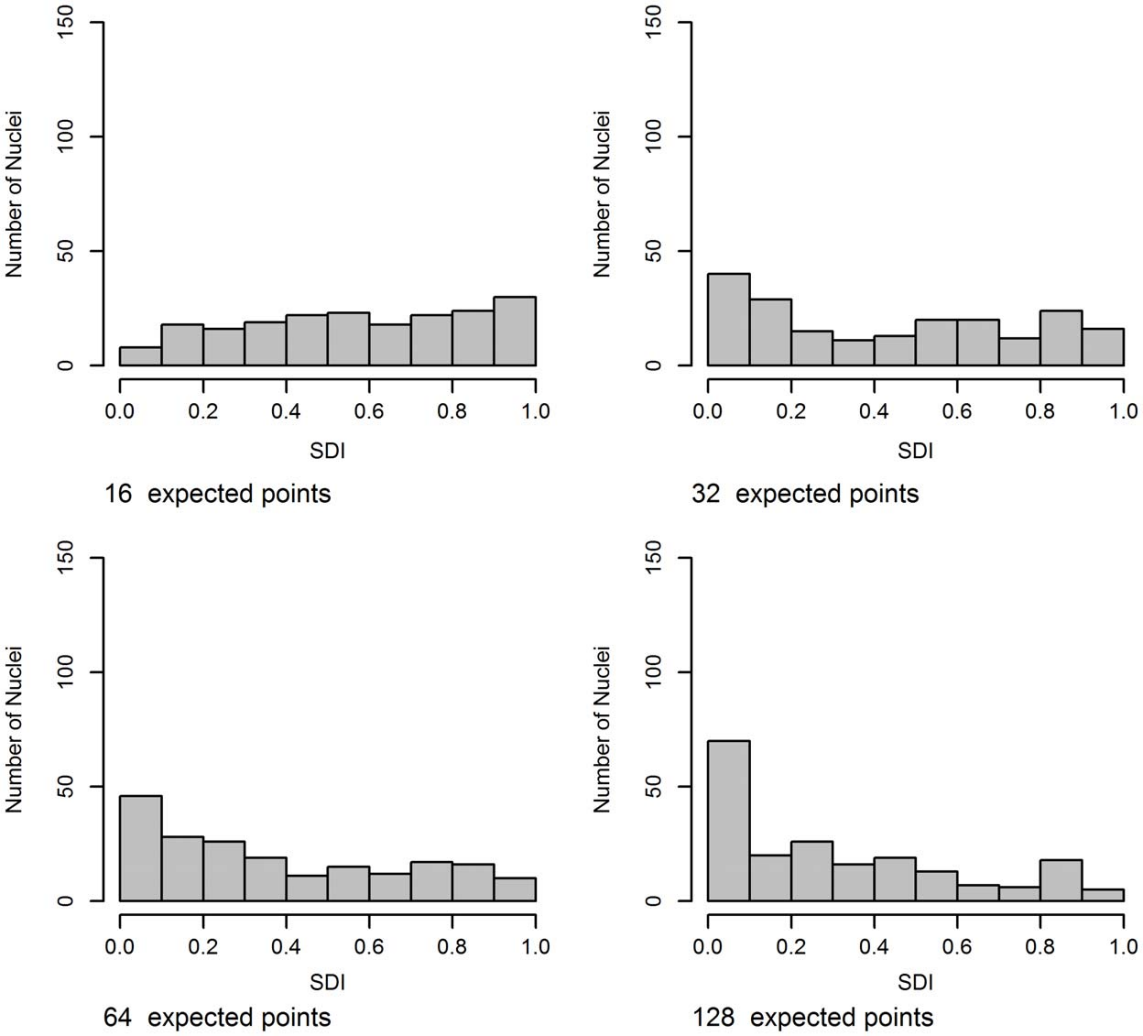
Histograms of SDI using F-function for the center alternative for the thick dataset with increasing number of expected points. The performance of SDI is similar, despite the difference in boundary shape.

### Testing Different Methods

The aim of the following experiments is to investigate the ability of methods proposed in the literature to identify potential non-CSR processes from point patterns that consist of a small number of points. In particular we look at a number of ways of using the F-function [12] that deploy different strategies to aggregate information over replicates that were described previously. The approaches are denoted *Individual F-function Test, Aggregate-Distance F-function Test* and *Aggregate-SDI*. The latter approach being based on the *Spatial Distribution Index* [13]. The first approach deploys the F-function Test (this and the following approaches are described in more detail in Materials and Methods) on each object individually. The Aggregate-Distance F-function Test standardizes the scale of all instances by dividing inter-point distances measured by the maximum inter-point distance within the boundary. An F-function Test is performed on the aggregated F-functions from all the target point patterns using the aggregated null distribution from each instance. For Aggregate-SDI, the SDI for each instance is measured individually as described in Materials and Methods.

#### Analysis of MRC5 data

For the Individual F-function Test, Figure 7 shows the F-function with a 95% confidence envelope [4] for three cells. In addition, the 2D projection of each raw image, and the corresponding processed data, is included. It is not at all clear, from inspection, what kind of pattern is present in these individual images. The key point of this paper is that this is true also of routine application of spatial analysis and aggregation. The leftmost cell does not exit the confidence envelope, [4], so there is no evidence to reject the hypothesis that the spatial configuration of PML compartments is CSR. The remaining two graphs show examples of a pattern that is more clustered than would be expected under CSR and a pattern that is more regular than would be expected under CSR, respectively. For the entire dataset we are able to reject the null hypothesis for 42 out of 50 cells, at the 95% significance level. This is close to expected false positive rate for a test at this significance level. However other real examples do not lead to such strong conclusions where as many as half the population may fail to reject CSR.

The Aggregate-Distance F-function Test fails to reject the null hypothesis for the replicate data. Scaling by the maximum distance between points on the boundary appears to be the incorrect method for aggregation. The distance distribution is not commensurate over boundaries with different shapes. Consider the sphere in Figure 3 and the ellipse on the top row in Figure 3. Scaling by the maximum length will result in the elliptical shape to be much thinner in both the z and y axes.

This form of normalization results in a larger spread in the confidence envelope. This is demonstrated by performing a modified Individual F-function Test where we replace the null confidence envelope with the aggregated null confidence envelope In this case fewer cells are found to reject the null, a total of 26 out of 50. Figure 8 shows the normalized F-Function corresponding to the same cells as Figure 7.

Aggregation using the Spatial Distribution Index results in a rejection of the null hypothesis of CSR at the 95% significance level. A histogram of the SDI values is shown in Figure 9 which demonstrates clear preference for high SDI and to a lesser degree a preference for low SDI. Under the hypothesis of CSR, this histogram should be approximately consistent with a uniform distribution that is the height of each bar in the histogram should be approximately equal. The preponderance of small and large distances, associated with the taller bars in Figure 9, suggests that there are two distinct types of departure from CSR. While this is potentially informative, it does not reveal much about the non-CSR nature of the generating process.

Of the three approaches, only the aggregated methods provide an unequivocal statement about the existence of non-CSR point processes determining the location of the PML bodies. Unfortunately they give conflicting results, although it appears likely that aggregating by distance is a less powerful approach than aggregating SDI values. In the next section we investigate synthetic datasets and show circumstances in which Aggregate-SDI can potentially miss non-CSR structure.

**Figure 14:**
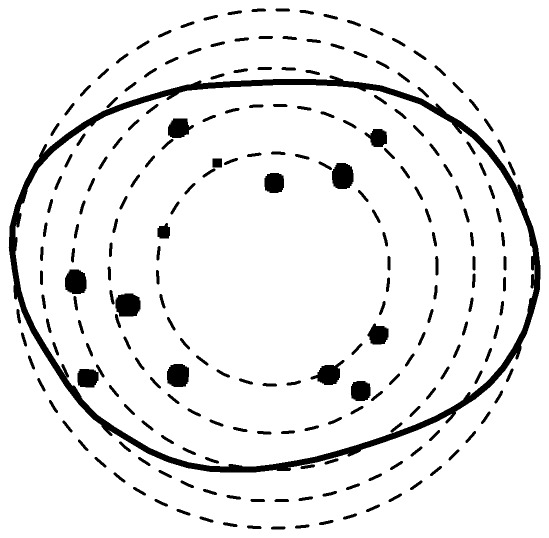
Radial Analysis of PML Bodies in a 2-D projected nucleus. A binning strategy that measures only distances from the center of gravity may not respect the nucleus envelope. The dotted circles are bin boundary locations where each bin has equal area. Bin regions can occur outside of the nucleus envelope, which can affect the analysis.

**Table 2:** Primary antibodies.

Compartment	Species	Specificity/concentration of antibody	Source	Titre
PML nuclear body	Rabbit	All isoforms of human PML protein. (Borden et al 1995).	Pure bleedout rabbit serum.	1 in 200
Nucleolus	Mouse IgG1	Nucleophosmin (B23) C-terminus of B23 in human, mouse and rat. 0.5 µg/µl.	Zymed Laboratories Inc. (32-5200).	1 in 50
Lamin B	Goat IgG	Lamin B of mouse, rat and human. 0.2 µg/µl.	Santa Cruz Biotechnology Inc. (Lamin B (M-20): sc-6217)	1 in 100

**Table 3:** Secondary antibodies.

Species	Specificity/concentration of antibody	Fluor conjugate	Source	Titre
Donkey	Rabbit IgG, 1.5 mg/ml	FITC	Jackson ImmunoResearch Laboratories, Inc.	1 in 200
Donkey	Mouse IgG, 1.4 mg/ml	Cy3	Jackson ImmunoResearch Laboratories, Inc.	1 in 200
Donkey	Goat IgG, 1.5 mg/ml	Cy5	Jackson ImmunoResearch Laboratories, Inc.	1 in 200

#### Analysis of synthetic data

For the synthetic data case, all the instances have spatial point patterns that have non-CSR structure, namely Center, Boundary and Polar. The three analysis methods described above are deployed to assess their capability to identify non-CSR structure. The experiments are repeated with an increasing expected number of points, namely 16, 32, 64 and 128. This corresponds to compartments that manifest an increasing number of objects per nucleus. In general, the higher the average number of points the easier it should be to identify the existence of non-CSR preferences. The main result we see is that the heterogeneity of shape is sufficient to compromise the efficacy of the procedures.

The results for the individual F-function Test and modified individual F-function Test are shown in terms of power. Recall that the power of a hypothesis test is the probability of rejecting the null hypothesis when it is false. In these experiments the null hypothesis is CSR and the null is always false (all our point patterns are not generated from a CSR process). So the power in these experiments equate to the proportion of instances that reject the null hypothesis. Figure 10 shows the result for the Individual F-function Test. Although we do see improvement in performance as the average number of points increases the power is very low for patterns with fewer expected points. There is a good chance that using this test would miss these types of spatial preference. Notice that in Figure 10, even with an average of 32 points we could miss a relevant spatial preference approximately more than half of the time. With real data, this would represent a tragically missed opportunity. Figure 10 also shows the Boundary alternative appears to be difficult to detect, especially for the thin dataset. We speculate that for any position within the synthetic boundary its closest point on the boundary will often be located above or below, hence the x-y (see Figure 3) component of interpoint distances of the Boundary process are more similar to CSR than other alternative processes.

Deploying *Aggregate-Distance* F-function Test fails to reject the null on for all examples from the thick and thin datasets.

The modified Individual F-function Test is examined in two ways. First, using a null hypothesis derived from the null distributions of all instances from the same shape class and second, all null distributions are aggregated from all instances, see Figure 11. In both cases Figure 11 shows that increasing aggregation in general degrades rather than enhances the performance. This is indicative of a test procedure fundamentally incapable of identifying the alternative process. Note that while there are other interesting features in these figures, they do no add anything useful to our main argument, namely that standard procedures are not always effective at identifying non-CSR processes.

Aggregation using the SDI values is far more successful than distance normalization. Table 1 shows, D, the summary statistic from the SDI, and its corresponding p-value for each of the alternative spatial point patterns, with increasing average number of points. There is a general trend that the p-value decreases as the expected number of points increases, increasing the number of samples would make this trend clearer. For Polar and Center we can reject the null in most cases, but for a low average number of points there is a possibility of missing these specific spatial preferences. In this experiment Polar process with 16 expected points was indistinguishable from CSR. We failed to reject the null for the boundary spatial point process for most cases. Both Figures 12 and 13 show histograms of the SDI values for the center spatial point process case, where we can clearly see the non-uniformity becoming more pronounced as the expected number of objects increases.

## Discussion

In a number of scenarios involving investigating a possible connection between the spatial preference of nuclear objects and nuclear function, the number of nuclear objects tends to be fairly small. In these circumstances we must be cautious about accepting the null hypothesis that the compartments of interest exhibit no interesting spatial preference. Investigating the spatial structure over a population of cells is likely to improve the power of tests for such preferences. Simple transformations of the spatial point patterns to make them commensurate yields the potential to reason about a collection of images, thereby increasing the power of such tests. However the method of aggregation used *must* be meaningful and not induce artifacts into the spatial point pattern. One possible approach is to build Aggregate Maps [24], [25]. This method for exploratory data analysis provides an interpretable representation of the spatial preferences of compartments within the nucleus derived from replicated images. Alternatively, investigating at the population level of individually analyzed cells can be achieved by deploying measurements such as SDI, [13]. Our experiments using the F-function found that distance normalization was ineffective for aggregation. This does not mean that distance normalization should be avoided altogether. It is a matter for future research to determine its effectiveness in aggregating spatial histograms and for other problems such as determining co-localization.

Radial analysis procedures, popular in the literature, involve first identifying the center of the nucleus, [6]. This is typically held to be located at the center of gravity of the nucleus, since there are no biologically meaningful locations within the nucleus that can be used. There at least two classes of radial analysis procedure. In the following we briefly describe and critique each class. In the first class, distances of target compartments are measured from the center of gravity to produce a histogram. One general issue arises from the arbitrary choice of bin width. A second issue relates to the fact that that the bins boundaries may not respect the nucleus boundary. Figure 14 shows an example where bin regions can be outside the nucleus envelope. In this example each bin has equal area, however the effective area, that is to say the area of a bin that an object can legitimately be placed, may not be equal. This makes testing for CSR harder than simply testing for uniformity across bins.

The second class of radial analysis procedure deals with this problem by allowing the shape of histogram boundaries to more closely follow the nuclear envelope itself, [9]. Other binning strategies have been used, especially in 2D, where the bins have been manually defined to delineate regions [26]. In all of these approaches there is still the problem of how to determine what the corresponding bin shape and location is across replicates especially when their boundaries differ.

While popular, it should be clear from the critique above that radial analysis procedures have significant shortcomings. Consistent with the central theme of this paper, the problem of aggregation across nuclei, extending radial analysis to multiple images is not at all straightforward and the issues described above are amplified. Hence we do not consider radial analysis any further. However we note that methods for binned aggregation would be a fruitful line for further research.

The take-home message is thus twofold. First, cell biologists are missing potentially interesting spatial relationships because of an over-reliance on standard and aggregated test procedures for CSR. Second, the analysis should not finish with a simple decision about CSR. Rather, more detailed analysis should be pursued for non-CSR patterns, but this is most sensible if the initial test procedures are performed carefully.

## Materials and Methods

### Synthetic Shape Boundary

Our synthetic boundary is constructed from a piece-wise function of ellipsoids,
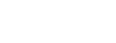
The shape parameters 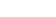 are used to define an ellipsoid fragment for each octant, 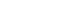. In order to maintain continuity at the boundaries of each octant we restrict the shape parameters in the following way,



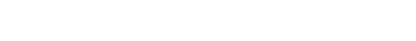
Similarly, the values 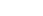 and 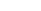 are assigned to the shape parameters 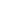 where we use the *y*-coordinate and the *z*-coordinate respectively.

Figure 3 shows four shape boundary classes, on the left, top row a sphere defined using,
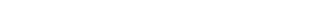
On the bottom row to the right, a cell-like shape extruded along the positive x-axis and flattened along the negative z-axis is defined using,



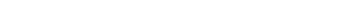
Our simulations will look at two shape collections, denoted the *thick* and *thin* datasets. The thick dataset consists of instances drawn from all four nucleus shape classes shown in Figure 3. These four shapes are defined by the value of 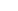,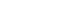, this corresponds to the lengthening of the boundary along the positive x-axis and 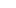,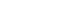 flattens the shape along the negative z-axis. All the remaining parameters are equal to 1.

The thin dataset consists of flatter shapes, these are created by setting 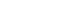 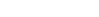 The remaining parameters are identical to the thick dataset. Shape boundaries with this character are rather similar to the real MRC5 dataset, see Figure 4. However we prefer to explore performance over a variety of cell shapes to demonstrate the generality of our conclusions.

Fifty instances of each of these shapes are generated by perturbing the parameters specifying each shape as follows. Consider the parameter 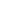. To generate a simulated realization 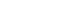, where 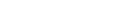. Here 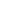 is a scaling parameter selected to induce variability and to avoid pathologies. All parameters are simulated similarly using the same scaling parameter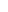. There is some arbitrariness in the choice of this parameter, our choice of 0.2 is based on trial and error to provide convincing shapes.

### Generating Alternatives to CSR

Alternatives to CSR are generated by thinning a realization from a CSR process [12]. For a shape boundary *S*, first a point *x* is drawn from a CSR process that has as its support the volume enclosed by *S*. The probability the point *x* is then retained is,
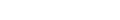
where *k* is a constant (set to 1 in the experiments) and the function, 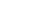 is the Euclidean distance of the point *x* from locations specified on the shape S. For the polar case we first specify two end-points 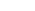 which are the located at the intersection of the shape with the *x*-axis, see Figure 5.


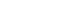
 is the minimum distance of the point *x* from the endpoints using only the *x*-axis component. For the center process,


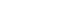
, measures the distance from *x* to the mean of the boundary (marked by the letter *c* in Figure 5) of *S*.


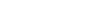
 is the distance from *x* to the closest point on the boundary of *S*.

The size of a realization that has an expected number of points 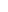 is determined by sampling from a Poisson distribution with mean 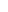. Note that in this case we use a truncated Poisson distribution to avoid empty or sparsely populated (less than 5) cells, and it is with respect to this distribution that expectation is taken. The procedure described above is then repeated until we have the required number of points.

The value of *k* determines the degree of aggregation, using a large value for *k* would result in highly aggregated points that should be easy to distinguish from realizations from a CSR process. We set the value of *k* to 1 for all spatial point patterns. The choice is made in order to produce spatial point patterns that could not be trivially detected as realizations from non-CSR spatial point processes and indeed could not be seen to deviate from CSR by visual inspection.

### Calculating the F-Function

The F-function, or empty space function, 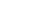 is defined as the probability that a point is within distance 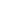 of a target point pattern. We estimate 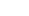
by following the Monte Carlo approach proposed in [13], as follows. For a shape with boundary *S*, 10,000 points are drawn from a CSR process that has as its support the volume enclosed by *S*. The shortest distance from each of these points to the target point pattern is measured. 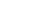 is estimated to be equal to the fraction of points that have a distance smaller than r to the target point pattern.

Note, this function is affected by the shape of the boundary and in general corrections are applied to handle edge effects. However, testing against CSR realizations using the same boundary allows us to ignore edge effects [27].

### Performing the F-function Test

The F-function Test is a hypothesis test in which we define the null distribution to be that the target points have been generated by a CSR process. The test deploys the F-function where a confidence envelope [4] is defined, should the target pattern’s F-function exit the confidence envelope the hypothesis is rejected.

The null hypothesis is constructed by generating the F-functions of 500 CSR realizations that have the same pattern size as the target point pattern. The envelope is constructed by considering all distances, *r*, in turn. For each *r*, the 500 values of 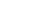 from the null realizations are used to define a 95% two-tailed confidence interval. Examples of the confidence envelopes can be seen in Figure 7.

### Spatial Distribution Index (SDI)

The Spatial Distribution Index, [13], is designed to aggregate information regarding the spatial configuration of point patterns. This description follows [13] including the setting of parameters (in particular the number of the Monte-Carlo iterations). For simplicity of exposition we describe SDI using the F-function, although it should be noted that other functions can be used.

The approach to calculating SDI can be conveniently split into two parts. First the F-function of the target point pattern, denoted 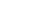, is compared to an average F-function for a CSR process, 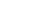 as follows.


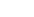
 is the mean of the F-functions of 500 realizations of a CSR process, each having the same number of points as the target pattern. The dissimilarity between 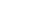
and 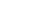, denoted 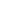, is defined to be the largest signed separation between the two curves.


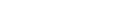
, where 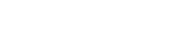.

Next the value 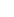 is transformed such that if the target process is CSR, then the transformed value will be uniform over 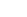. This transformation is achieved by recording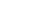, the dissimilarities between 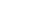 and the F-functions of a further 500 realizations of the CSR process described above. The SDI value is defined as the fraction of 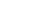 that are smaller than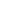.

The hypothesis test that the spatial point patterns from multiple cells follow a CSR process is performed by testing the distribution of SDI for uniformity using a two-sided Kolmogorov-Smirnov test at 5%.

### Confocal Laser Scanning Microscopy (CSLM)

In CSLM, the compartments of interest are stained such that they fluoresce at the focal point of the laser light, which is scanned across the entire volume of the sample nucleus revealing compartment locations. Staining is often achieved using indirect immunofluorescence in which a fluorescent dye is attached to antibodies that have been selected against proteins within the target functional compartment. The output from these analyses is a stack of intensity images, representing the 3D volume of the nucleus with locations of target compartments. In each image, the intensity at each voxel serves to reveal in part the relative concentration of a particular antibody and by inference target protein at that location. However as immunostaining is indirect, any increased intensity can also arise from an increased availability of accessible epitopes. This issue is usually resolved by bespoke image processing schemes intended to distinguish compartments from background. A discussion of these issues, and a particular procedure, is given in [2]. The technical details describing the methods for cell staining and CSLM follow in the next section.

### MRC5 Cell Line

Cells were cultured in T25 or T75 flasks at 37°C and with 5% CO_2_. Cell lines and corresponding culture media used, were as follows. MRC5 human Caucasian male foetal lung fibroblasts, a normal diploid human cell line (ATCC), were cultured in RPMI 1640 supplemented with 10% FCS, 2 mM L-glutamine, and Penstrep (50 IU/ml penicillin, 50 µg/ml streptomycin), (all Invitrogen).

#### Cell staining and confocal microscopy

MRC5 human foetal lung fibroblasts were plated onto glass coverslips in six well plates, and incubated at 37°C and with 5% CO_2_. After 48 hours cells were fixed in 4% paraformaldehyde in PBS for 10 mins, then permeabilised in 0.5% Triton X-100 in PBS for 20 mins. Cells were concurrently incubated with primary antibodies against nuclear antigens for 30 mins at 37°C. After washing in PBS they were incubated with appropriate secondary antibodies for 30 mins at 37°C in the dark. Z stacks of nuclei were captured using a Zeiss LSM 510 confocal microscope with a Zeiss Plan Apochromat 63× oil immersion objective with a numerical aperture of 1.4, and a zoom of 3.4. Z-stacks typically consisted of about 20 sequential slices of 250×250 or 300×300 pixels (where 1 µm = 12 pixels) captured at 0.4 µm intervals through the nucleus.

#### Indirect immunofluorescence

PML was identified using a rabbit anti-PML antibody (Borden et al 1995) and detected with an FITC-conjugated donkey anti-rabbit secondary antibody (Jackson ImmunoResearch Laboratories, Inc.). The edge of the nucleus was delineated with a goat anti-Lamin B antibody (Santa Cruz Biotechnology Inc.) and detected with a Cy5-conjugated donkey anti-goat secondary antibody (Jackson ImmunoResearch Laboratories, Inc.). The nucleoli were stained using a mouse anti-Nucleophosmin/B23 monoclonal antibody (Zymed Laboratories Inc.) and a Cy3-conjugated donkey anti-mouse secondary antibody (Jackson ImmunoResearch Laboratories, Inc.). Slides were mounted in 0.25 µg/ml DAPI in glycerol/PBS and Citifluor AF1 antifade, which allowed nuclei to be located for imaging. Details of antibodies are given in Table 2 and 3.
